# Relationship Between the Eruption of Third Molar Teeth and the Growing Status of the Assamese Inhabitants of a North-Eastern State of India

**DOI:** 10.7759/cureus.21044

**Published:** 2022-01-09

**Authors:** Putul Mahanta, Kahua Das, Himamoni Deka, Bharati Basumatari, Ranjumoni Konwar, Plabita Mazumder, Madhab C Rajbanshi

**Affiliations:** 1 Forensic Medicine and Toxicology, Assam Medical College and Hospital, Dibrugarh, IND; 2 Physiology, Tezpur Medical College and Hospital, Tezpur, IND; 3 Anatomy, Gauhati Medical College and Hospital, Guwahati, IND; 4 Radiology, Fakhruddin Ali Ahmed Medical College (FAAMC) and Hospital, Barpeta, IND; 5 Dentistry, Assam Medical College, Dibrugarh, IND; 6 General Surgery, Tezpur Medical College and Hospital, Tezpur, IND

**Keywords:** physical maturity, eruption of teeth, nutritional status, third molar teeth, body mass index

## Abstract

Objectives

Even though permanent teeth eruption time may vary, the tooth eruption stage represents a critical developmental milestone, which may help in the monitoring of the child's growing status towards adolescence. This paper aims to evaluate the relationship between the third molar eruption (TME) and investigate any possible association with the body mass index (BMI), which is used to monitor the growth of children and adolescents.

Methods

We included 1060 student participants aged 14 to 26 years from selected educational institutions in Assam for this study. We performed a conventional clinical dental examination to determine the stages of TME. Moreover, to calculate the individual height and weight, we have carried out a thorough physical examination to assess the BMI and rule out any visible congenital developmental anomalies.

Result

The overall mean age of the status of no eruption (NE), incomplete eruption (IE), and complete eruption (CE) categories of the third molar in the current research were 17.39±2.273, 18.67±2.282 and 20.33±2.566 years, respectively, and the difference in mean age was statistically significant (p-value <0.05). Among the 1060 participants assessed, 163 (15.38%) were underweight, 625 (58.97%) belonged to the average category, 207 (19.52%) were at risk of being overweight, and 65 (6.13%) were obese. The CE in both average and overweight categories was 109 (17.4%) and 37 (17.9%). Similarly, the NE status of the third molar in the underweight and obese category was 120 (73.6%) and 39 (60.0%), respectively. These differences in the frequency of TME in different BMI categories were found statistically significant (p-value <0.05).

Conclusion

The result shows a substantial relationship between the mean age of eruption of third molar teeth and BMI among children and adolescents in 14 years to 26 years in the Assamese population. Further, the mean age can be used in forensic age determination.

## Introduction

The teeth go on to grow during teenage years and early adulthood. Teeth eruption is a substantial effect that is related to physical growth and is used as a measurement for children's maturity. It provides a biological marker for the age assessment of adolescence and adulthood. The parents often convey their fear concerning the timing of the teeth budding in their child's jaw.

It is mentioned in the literature that the age of the tooth eruption varies physiologically because of factors like gender, hereditary, nutritional and geographic influences [1]. Hence, while a method to estimate the age is developed from third molar eruption (TME), genetic, nutritional, and geographical factors are to be considered due to their influence on the eruption process of teeth [2]. In addition, for treatment planning in dentistry and community medicine, accurate knowledge of the timing of eruption of permanent teeth in children and adolescents is also essential [3].

The eruption process of the third molar is related to the person's BMI status, and the overweight group of children affects their early eruption [4]. Similarly, chronic malnutrition during childhood results in a delayed eruption [5], like the way obesity helps in the early eruption [6], though a study contradicts this result [7].

Thus, nutritional status plays a constructive part in accelerating tooth eruption stages. Therefore, an eruption of teeth is related to the somatic growth of an individual. Accordingly, poor nutrition during the growth period delays the eruption process in temporary and permanent teeth, which causes congenital anomalies and poor oral health [5, 8-11].

The age and sequence in which the TME occurs are noteworthy in developing children's teeth, jaws, and overall growth [12]. Further, TME is the only available biological marker for determining a person's age in adolescence, as all other teeth have already completed their eruption process [13]. The age thus determined can be used to establish an individual's identity.

Moreover, the eruption time of the teeth determined from a region can't be generalized to other populations as it varies significantly in eruption and even the BMI [14].

No matter what of these outcomes, there is a scarcity of databases for evaluating the age of the TME which monitor growth. Although representing an essential contribution to estimate the age of TME and its relationship with nutritional status, the different studies performed do not resolve the medical professional's dilemma in this relevance.

Hence, this research was conducted to evaluate the average eruption age of third molar teeth and examine its relation to BMI, in order to monitor the healthy development of children and young adolescents.

## Materials and methods

Our research is cross-sectional descriptive-analytical research that was performed over the three medical colleges and hospitals and three different educational institutions from three different districts of Assam from July 2014 to July 2018. We have selected the educational institutions purposively. The reference population for this study was the students of Assam, a north-eastern state of India, aged between 14 years to 26 years.

A total of 1060 subjects with known chronological age were included in this study and a conventional dental and physical examination was done on the subjects.

While collecting the data, specific criteria for inclusion of the participants were considered that are as follows: Healthy subjects born with no history of surgery or trauma involving the posterior quadrants of both jaws; subjects who were non-syndromic (Alpert Syndrome, ectodermal dysplasia, Hunter Syndrome, etc.) and with no systemic disorder (Hyperparathyroidism, celiac disease, musculoskeletal diseases, Vitamin D resistant rickets, etc.), free from any known and visible congenital disorders and growth retardation and the samples free from diseases of teeth that may retard the process of growth and normal developments of the teeth.

However, the subjects excluded were those with impacted third molars, family history of known congenital disorders, the known congenital absence of the third molar, the history of third molar extraction, hypothyroidism and Down’s syndrome. The reason for excluding those cases is to have an exact result on the objective of the study. The insufficient space often causes the tooth impaction for eruption and is mainly encountered in the case of third molar eruptions. Hence patients with impacted third molars were not included in the present study, which was assessed clinically.

Out of seven, only three authors have assessed the stages of TME by conventional clinical dental examination. If any part of the crown was evident through the oral mucosa, a tooth was considered as having erupted. As per the stages of the eruption of the third molar based on the clinical dental examinations, the patients were categorised as no eruption (NE) status if the third molar is not visible in any of the quadrants of the jaw, incomplete eruption (IE) status if any of the third molars have visibly erupted but not all four; and complete eruption (CE) status if all four third molars have erupted at the time of the study. We have found the inter-observer agreement as 100% (Cohen's kappa) in a sample of 106 (10% of the total sample) selected individuals due to the apparent criteria for determining tooth eruption of the present study. Tooth calcification stages could not be assessed as the radiographic examination of the tooth was not undertaken in the present study.

Thorough physical examinations of the participants were carried out to calculate the individual height and weight to assess the BMI and rule out any visible congenital developmental anomalies.

The current study has compared the participants' TME status with chronological age, gender, and BMI to assess any potential relationships. Also, the TME status was further classified as “Not erupted” if none of the third molars has erupted and “Erupted” if any of the third molars have erupted. We have also performed the binary logistic regression analysis to assess the contribution of the dependent variables in the TME.

Statistical analysis

Descriptive statistical methods, viz., frequency, percentage, Mean±SD and Standard Error of the Mean (SEM) were computed to study the distribution of the variables under study. Chi-square test, student's t-test and one-way analysis of variance (ANOVA) were used to test the significant differences among different categorical and continuous variables, respectively. Binary logistic regression analysis was performed to identify potential factors affecting the TME of the participants. The data analysis was done by using Microsoft Excel (Microsoft Corporation, Redmond, WA) and the Statistical Package for Social Studies (SPSS) version 22 (IBM Corp., Armonk, New York). A p-value <0.05 was considered significant.

The Institutional (Human) Ethics Committee (IHEC) of Tezpur Medical College has given the ethical approval vide ref: IHEC No- 02/IEC/IMC/14 to conduct this study. Before data collection, informed consent was obtained from the participants.

## Results

Out of the total 1060 participants, 642 (60.56%) were males, and 418 (39.43%) were females. The data analysis shows in Table 1 the frequency of the stages of the TME with the chronological age. All 38 participants of age 14 years showed NE while three (2.2%) participants showed CE at a very early age of 15. The differences in the frequencies of TME at different chronological age groups were found statistically significant (p-value < 0.001).

**Table 1:** Dental eruption status with chronological age *Statistically significant

Variables		Total	Complete eruption (CE)	Incomplete eruption (IE)	No eruption (NE)	p-value
Chronological age in years	14	38	0 (0.0%)	0 (0.0%)	38 (100.0%)	< 0.001*
	15	139	3 (2.2%)	19 (13.7%)	117 (84.2%)	
	16	140	6 (4.3%)	24 (17.1%)	110 (78.6%)	
	17	190	21 (11.1%)	47 (24.7%)	122 (64.2%)	
	18	87	8 (9.2%)	29 (33.3%)	50 (57.5%)	
	19	129	25 (19.4%)	35 (27.1%)	69 (53.5%)	
	20	144	31 (21.5%)	40 (27.8%)	73 (50.7%)	
	21	100	32 (32.0%)	27 (27.0%)	41 (41.0%)	
	22	40	12 (30.0%)	13 (32.5%)	15 (37.5%)	
	23	20	14 (70.0%)	3 (15.0%)	3 (15.0%)	
	24	11	5 (45.5%)	6 (54.5%)	0 (0.0%)	
	25	11	9 (81.8%)	0 (0.0%)	2 (18.2%)	
	26	11	6 (54.5%)	2 (18.2%)	3 (27.3%)	

The overall mean ages of NE, IE, and CE categories of the present study were 17.39±2.273, 18.67±2.282 and 20.33±2.566, respectively and were found to be statistically significant (p-value < 0.001 for one-way ANOVA). The differences in the mean age of individuals among males and females with NE status were statistically significant (p-value < 0.001) and are shown in Table 2.

**Table 2:** Ages at third molar eruption (TME) in male and female *Statistically significant; Figure in the brackets is the SEM (Standard Error of the Mean).

Status	Overall		Male		Female		p-value
	No.	Mean ± SD	No.	Mean ± SD	No.	Mean ± SD	
No Eruption (NE)	643	17.39 ± 2.273 (0.090)	371	16.92 ± 2.138 (0.111)	272	18.02 ± 2.303 (0.140)	< 0.001*
Incomplete Eruption (IE)	245	18.67 ± 2.282 (0.146)	163	18.55 ± 2.430 (0.190)	82	18.90 ± 1.948 (0.215)	0.249
Complete Eruption (CE)	172	20.33 ± 2.566 (0.196)	108	20.29 ± 2.547 (0.245)	64	20.39 ± 2.616 (0.327)	0.799

Among the 1060 participants assessed, 163 (15.38%) were underweight, 625 (58.97%) belonged to the normal category, 207 (19.52%) were overweight, and 65 (6.13%) were obese. The frequency of complete TME is lower in the underweight and obese categories. The differences in TME among different BMI groups are significant (p-value <0.021), as shown in Table 3.

**Table 3:** Relation of third molar eruption with different BMI group *Statistically Significant; BMI- Body Mass Index

Variables		Total	Complete Eruption (CE)	Incomplete Eruption (IE)	No Eruption (NE)	p-value
BMI	Underweight (≤ 18.5)	163	17 (10.4%)	26 (16.0%)	120 (73.6%)	0.021*
	Normal weight (18.5 - 24.99)	625	109 (17.4%)	148 (23.7%)	368 (58.9%)	
	Overweight (25.0 - 29.99)	207	37 (17.9%)	54 (26.1%)	116 (56.0%)	
	Obesity (≥ 30)	65	9 (13.8%)	17 (26.2%)	39 (60.0%)	

Although it was observed that the mean age of eruption increased with increasing BMI indicating delayed eruption in obese children, however, the age-stratified analysis revealed no statistically significant association between TME status and BMI in different chronological ages (p-value >0.05).

The binary logistic regression was used to assess the potential factors affecting the TME. The unconditional simple logistic regression revealed that individuals with higher chronological age have a significantly higher chance of TME (Crude OR = 1.394, 95% CI:1.316-1.476). Similarly, in our study, it was found that females had lower odds of TME as compared to males (Crude OR = 0.735, 95% CI=0.570-0.948). Also, underweight (Crude OR = 0.513, 95% CI: .350-.753) individuals had almost 0.5 times lesser odds of TME as compared to those with normal BMI. Multiple logistic regression models also predicted age and sex as the independent factors significantly affecting third molar eruption among the study participants (Table 4).

**Table 4:** Crude and adjusted odds ratios of different independent variables with third molar eruption (TME) *Statistically Significant

Variables	Crude OR	95% CI (Lower-Upper)	Adjusted OR	95% CI (Lower-Upper)
Chronological age	1.394*	1.316-1.476	1.416*	1.334-1.503
Gender				
Male	1	-	1	-
Female	0.735*	0.570-0.948	0.569*	0.429-0.755
BMI				
Normal	1	-	1	-
Underweight	0.513*	0.350-0.753	0.699	0.460-1.063
Overweight	1.123	0.818-1.543	1.292	0.915-1.825
Obese	0.955	0.567-1.608	1.178	0.675-2.065

## Discussion

The current result revealed the earliest TME at 15 years, with an average range of 15-25. Some studies support the third molar eruption during 15-25 years [15,16]. Nevertheless, these distinctions of mean age of CE amongst both genders were statistically insignificant, as uncovered by some other studies [15-18]. These findings may suggest that the third moral formation is a process associated with the beginning of puberty.

Another study [18] supports the overall mean age of complete TME as 20.33 (± 2.566) years in all quadrants. Although the mean age differences between males and females were statistically insignificant, it was observed early in the male with the mean age of 20.29 (± 2.547) years compared to the mean female age of 20.39 (± 2.616) years, a finding in accordance to some studies [15,16]. Overall, boys were found to have an earlier eruption of permanent teeth than girls as males are reported to attain some of the tooth development stages earlier than females, as reported in some studies [19-21].

The present findings have shown a relationship between the CE of the third molar and BMI. We observed a higher trend of CE of the third molar in participants with 37 (17.9%) overweight and 109 (17.4%) in the normal BMI category. Also, a declining trend was observed in 17 (10.4%) cases in the underweight and nine (13.8%) cases in the obesity groups. Similar results were opined by a study [22] reflecting the association of BMI and CE of the third molar. A study has opined that the overweight group of children affects their early eruption [4].

The third molar's delayed, or NE status was also observed most in the underweight category for 120 (73.6%) cases and followed by the obesity group for 39 (60.0%) cases which are in support of a research outcome [5].

The differences in the frequency of TME in different BMI categories were found statistically significant (p-value < 0.021). Therefore, an eruption of teeth is related to the nutritional supplement to the individual and plays a positive role in hastening the eruption stages. This association of the present study agrees with the research outcomes [22-24] done in different places. However, BMI is not the only influencing factor on the eruption stages of teeth.

Limitations of the study

The dental age should be compared with radiological age and sexual maturity for age accuracy apart from physical maturity, which was not done in the present study. In addition, third molar mineralization was not evaluated, and the impaction was not assessed radiologically. Another limitation of this study was the orthopantomogram (OPG) or cephalometric radiograph, which visualizes the dental development clearly, but is not carried out here. Hence this study recommends a more extensive investigation to know the accuracy of the relationship among the variables mentioned.

## Conclusions

The current study demonstrates a significant association between BMI and dental growth among children and adolescents aged 14 to 26 years in Assam, India’s north-eastern state. The age of different eruption statuses of third molar teeth provides valuable data that can be utilized and applied in the age investigation and to monitor an individual’s probable growth pattern under investigation.

A more extensive study is suggested to see the correlation between BMI and dental eruption status and its variation among the different ethnic groups. Simultaneously, population-specific data are essential while using wisdom tooth eruption for growth monitoring as it varies from population to population.
